# Moderate Weight Loss Decreases Oxidative Stress and Increases Antioxidant Status in Patients with Metabolic Syndrome

**DOI:** 10.5402/2012/960427

**Published:** 2012-11-06

**Authors:** Maria Del Ben, Francesco Angelico, Roberto Cangemi, Lorenzo Loffredo, Roberto Carnevale, Teresa Augelletti, Francesco Baratta, Licia Polimeni, Pasquale Pignatelli, Francesco Violi

**Affiliations:** I Clinica Medica, Dipartimento di Medicina Interna e Specialità Mediche, La Sapienza Università di Roma, Policlinico Umberto I, Viale del Policlinico 155, 00161 Roma, Italy

## Abstract

*Background*. Oxidative stress is enhanced in metabolic syndrome (MetS) and believed to contribute to accelerated atherosclerosis. Weight loss is associated with lowered oxidative stress. *Methods*. We performed a cross-sectional study in 92 consecutive patients with metabolic syndrome and 80 without. A dietary intervention with moderately low-calorie diet (600 calories/day negative energy balance) was carried out in 53 of metabolic syndrome patients. Oxidative stress, assessed by sNOX2-dp and urinary 8-iso-PGF2*α*, and antioxidant status, assessed by serum levels of vitamin E and adiponectin, were measured before and after 6 months. *Results*. Serum vitamin E/cholesterol ratio was significantly lower in metabolic syndrome compared to controls (*P* < 0.001) and decreased by increasing the number of metabolic syndrome components (*P* < 0.001). After six months, 23 and 30 patients showed >5% (group A) or <5% (group B) weight loss, respectively. Urinary 8-iso-PGF2*α* (−39.0%), serum sNOX2-dp (−22.2%), adiponectin (+125%), and vitamin E/cholesterol ratio (+129.8%) significantly changed only in A group. Changes in body weight and in serum adiponectin were independent predictors of vitamin E/cholesterol ratio variation. *Conclusion*. Our findings show that in metabolic syndrome moderate weight loss is associated with multiple health benefits including not only oxidative stress reduction but also enhancement of antioxidant status.

## 1. Introduction

The metabolic syndrome (MetS) has emerged as an important cluster of metabolic and nonmetabolic risk factors for atherosclerotic disease associated with insulin resistance [1, 2]. MetS is associated with an increased risk for developing cardiovascular disease and type 2 diabetes mellitus [3]. MetS patients are three times as likely to have a heart attack or stroke compared with people without the syndrome and have a fivefold greater risk of developing type 2 diabetes [4–6].

Lifestyle modifications in the form of moderate caloric restriction, moderate increase in physical activity, and changes in dietary composition towards a less atherogenic, low-saturated fat, and prudent diet are the most common modalities for managing patients with MetS. Current guidelines on the management of the individual components of MetS emphasize that moderate weight loss should be a first-line therapy [7].

Oxidative stress is believed to play a major role in vascular disease, as it is implicated in the initiation and progression of atherosclerosis; it determines inflammation of the artery wall, which ultimately leads to macrophage infiltration and anatomical and functional changes of endothelial wall.

Oxidative stress is a typical feature of MetS and obesity [8–11] as shown by enhanced levels of urinary isoprostanes, a marker of oxidative stress and sNox2-dp, and a marker of activation of NOX2, the catalytic subunit of NADPH oxidase [12].

Weight loss has been associated with reduced oxidative stress and NOX2 activation which lead to an improvement of endothelial dysfunction [12]. 

Vitamin E is a fat-soluble antioxidant that inhibits lipid peroxidation [13]. Serum concentration of vitamin E (alpha-tocopherol) depends on the liver, which takes up the nutrient after the various forms are absorbed from the small intestine. Major sources of alpha-tocopherol in the diet include vegetable oils (olive, sunflower, and safflower oils), nuts, whole grains, and some green leafy vegetables [14].

Blood antioxidants, including vitamin E, are consistently lower in obese than nonobese adults and progressively lower with increases in BMI [15–19]. However, the relationship between vitamin E and oxidative stress in MetS as well as the relationship with its clinical features are still unclear. Moreover, so far, there is no information on the effects of weight loss on vitamin E blood levels. 

Aim of the present study was twofold. First, to evaluate serum levels of vitamin E in patients with and without MetS. Second, to assess changes of vitamin E and markers of systemic oxidative stress in MS patients adhering to a moderately calorie-restricted, Mediterranean-type diet during a 6-month study period.

## 2. Material and Methods

### 2.1. Patients

The cross-sectional study vas performed in 172 consecutive outpatients, aged 40–80 years, referred to our metabolic outpatient clinic at the Department of Internal Medicine of the University Hospital in Rome. MetS was diagnosed in 92 patients following the recently harmonized criteria by major scientific organizations based on the concomitant presence of at least three of the following five clinical features: waist circumference (central obesity) ≥102 cm in men and ≥88 cm in women, fasting blood glucose ≥100 mg/dL, triglycerides ≥150 mg/dL, HDL cholesterol <40 mg/dL in men and <50 mg/dL in women, and arterial systolic/diastolic blood pressure ≥130/≥85 mmHg [4]. Eighty subjects not fulfilling the above criteria for MetS were considered as controls. The intervention study was carried out in 53 out of the above patients with MetS, who were willing to participate in the study. Patients had to meet the following criteria: no history and clinical signs of cardiovascular disease, autoimmune disease, acute inflammatory disease, or any severe disease shortening life expectancy, such as diagnosed cancer, chronic liver disease, and severe renal disease; no regular intake of antioxidant vitamins, statins, or other drugs with known antioxidant properties; and no current smoking. Clinical, nutritional, and biochemical characteristics of patients in the intervention study, as well as the results of weight loss on endothelial function, have been previously described [12].

Written consent was obtained from enrolled subjects and the study was conformed to the ethical guidelines of the Declaration of Helsinki; the ethical committee of our Institution approved the study.

### 2.2. Dietary Intervention

Participants were instructed to consume a moderately calorie-restricted diet during the 6-month study period. They followed a balanced low-calorie (600 calories/day negative energy balance), low-fat, high-carbohydrate diet (<30% of energy from fat, <10% from saturated fat and 55% from carbohydrate) under supervision of the study nutritionist. The goal was to achieve a weight loss of approximately 5% of initial body weigh over six months. Patients were asked to increase the consumption of fruit, vegetables, low-fat dairy products, poultry, fish, and whole grain products, to limit the intake of red meat and of high-fat dairy, to have only moderate amounts of sugars and foods containing added sugars, and to have olive oil as the only seasoning and cooking fat. They received sample menus, recipes, and instructional nutrition labels in order to achieve a better healthy food selection and a daily negative energy balance. The diet was distributed in 5 meals, with 2 breaks during the day. At baseline and after the diet period a weekly food frequency questionnaire was administered. All foods were self-selected and prepared by the subjects and no supplements were prescribed. Energy requirements were calculated at baseline by estimating basal metabolism and resting energy expenditure by the Harris-Benedict equation. Subjects were asked to maintain the same level of physical activity throughout the study period.

### 2.3. Blood Sampling Protocol

After overnight fasting and supine rest for at least 10 minutes, blood was withdrawn from an antecubital vein. Blood samples were taken into tubes containing EDTA and centrifuged at 3,000 rpm for 15 minutes to obtain plasma. Serum and plasma were divided into aliquots and stored at −80°C.

*Serum concentration of vitamin E* was measured by HPLC using tocopheryl acetate as internal standard. Reagents included HPLC-grade ethanol, methanol and hexane (E. Merck, Darmstad, Germany), and tocopheryl acetate (Sigma Chemical, St. Louis, MO). A flow rate of 2.0 mL/min was used with a LC/233 Diode Array Detector (Restek Corporation PA, USA) set at 0.02–0.1 attenuation. Levels were expressed as the ratio between serum alpha-tocopherol concentration (*μ*mol/L) and serum total cholesterol concentration (mmol/L) [19, 20]. Intra- and interassay coefficients of variation were 6.2% and 7.2%, respectively.

*Urinary 8-iso-prostaglandin F2*α* (8-iso-PGF2*α**) was measured by a previously described and validated enzyme immunoassay method [21]. Intra-assay and interassay coefficients of variation were 2.1% and 4.5%, respectively.

*Serum levels of soluble *NOX2-derived peptide (sNOX2-dp) were detected by ELISA method as previously described [22]; intraassay and interassay coefficients of variation were 5.2% and 6%, respectively. Values are expressed as pg/mL.

*Adiponectin *serum levels were measured with a commercial immunoassay (Tema Ricerca, Italy). Intra-assay and interassay coefficients of variation were 6% and 8%, respectively. 

### 2.4. Statistical Analysis

Statistical analysis was performed using the SPSS statistical software version 13.0 for Windows (SPSS Inc., Chicago, Illinois). All variables were tested for normal distribution prior to analyses. Data are expressed as the mean ± SD for continuous variables. The correlation between variables was analysed with the Pearson test and the Cochran's linear trend test. Student's *t*-test for unpaired data was used for the comparison of mean values. Group comparisons were performed by use of analysis of variance (ANOVA). Proportions and categorical variables were tested by the *χ* ^2^-test and by the 2-tailed Fisher's exact method when appropriate. Multiple stepwise forward linear regression analyses were performed to identify the independent predictors of vitamin E/cholesterol ratio and vitamin E/cholesterol ratio changes after dietary intervention. To test the independent predictive role of vitamin E/cholesterol ratio for MetS, a logistic regression analysis was performed.

All *P* values are two tailed; a *P* value of less than 0.05 was considered statistically significant.

### 2.5. Sample Size Calculation

For the cross-sectional study, the number of MetS patients and controls was computed with respect to a two tailed Student's *t*-test for independent groups, considering (i) expected difference in Vitamin E/cholesterol ratio to be detected between patients and controls = 0.6 *μ*mol/mmol, (ii) standard deviations homogeneous between groups SD = 1.1 *μ*mol/mmol, and (iii) type I error probability *α* = 0.05 and power 1-*β* = 0.90. This resulted in *n* = 71 per group, which was increased to *n* = 80. For the intervention study, as previously reported [12], we hypothesized a rate ≥15% of patients with unsuccessful weight loss after diet (weight loss < 5%) and computed a minimum sample size of*n* = 15 patients for group to participate in the intervention study.

## 3. Results

### 3.1. Cross-Sectional Study

Clinical and biochemical characteristics of MetS patients and controls are reported in Table 1. As expected, patients with MetS had significantly higher prevalence of central obesity, high blood pressure, hypertriglyceridemia, hyperglycemia, and type 2 diabetes, as compared to non-MetS ones. Vitamin E/cholesterol ratio was significantly lower in MetS compared to control patients (4.5 ± 1.2 versus 5.2 ± 0.8 *μ*mol/mmol, *P* < 0.001).

**Figure 1:**
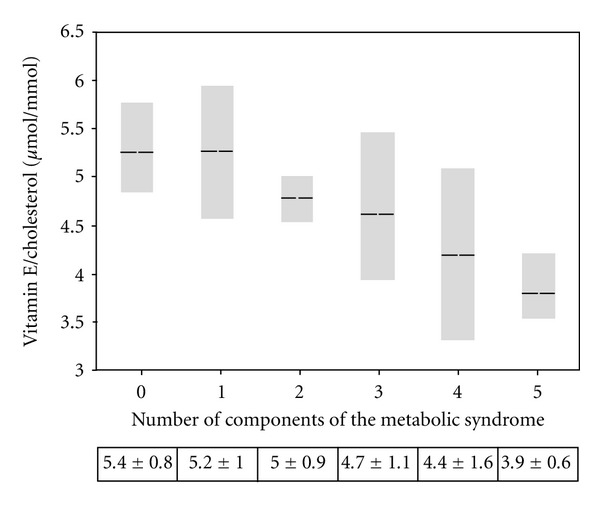
Mean serum vitamin E/cholesterol ratio according to the number of components of metabolic syndrome. Distribution of vitamin E/cholesterol (*P* for trend <0.001) according to presence of 0, 1, 2, 3, 4, or 5 components of metabolic syndrome. Box plots demonstrate median, 25th, and 75th percentile values. Cells report means ± SD.

In all subjects, vitamin E/cholesterol gradually decreased by increasing the number of MetS components (*P*_trend_ < 0.001) (Figure 1). 

As previous studies indicated that patients with vitamin E/cholesterol ratio <5 are at higher risk of vascular events [23, 24] we divided the entire population in two groups on the basis of vitamin E/cholesterol ratio < or ≥5 (Table 2). The prevalence of all the components of MetS was significantly higher in patients with vitamin E/cholesterol ratio <5. Finally, vitamin E/cholesterol ratio was found to be independently associated with MetS (O.R. : 2.6, 95% C.I. : 1.3–5.1, *P* = 0.006), after controlling for confounding factors.

### 3.2. Intervention Study

Patients included in the interventional study had clinical characteristics similar to those of the entire population except for the exclusion of diabetes (data not shown). During dietary treatment some patients adhered more closely to the prescribed diet and, after six months, achieved much better weight loss than others. Therefore, for the purpose of the present analysis, participants were divided in two groups according to body weight loss higher or lower than 5%, respectively. A good compliance to dietary recommendations was found in 23 subjects (group A), who lost 7% of their initial weight over six months, while 30 subjects (group B) showed no significant weight changes (+0.1%) (Table 3). Baseline characteristics were not significantly different between the two groups. At the end of the intervention period, the estimated total calorie intake of group A was 2156 ± 364, whereas that of group B was an average of 2870 ± 455. Details on nutritional changes as well as the effects of weight loss on endothelial function have been previously reported [12].

Major nutrient changes concerned dietary fat. In fact, in keeping with the study protocol, group A markedly reduced (−58.6%) dietary consumption of olive oil and seasoning fat, which led to an estimated decrease of vitamin E intake from 39.3 to 16.2 mg/day. Conversely, subjects belonging to group B showed no significant changes in dietary fat and vitamin E intake.

Baseline and after six months characteristics of patients belonging to group A and group B are reported in Table 3. In group A, a significant reduction of oxidative stress, as assessed by urinary 8-iso-PGF2*α* (−39.0%) and serum sNOX2-dp (−22.2%), was observed, together with an increase of serum adiponectin (+125%). In the same group, mean serum Vitamin E/cholesterol ratio increased significantly (from 4.8 ± 1.7 to 9.4 ± 2.9; *P* < 0.001), while nonstatistically significant changes were observed in group B (mean between groups difference +4.8 [95% C.I. 3.5 to 6.1]; *P* < 0.001).

At bivariate analysis performed in all subjects (Table 4), a strong statistically significant negative correlation was observed between plasma Vitamin E/cholesterol ratio changes and changes of body weight (*r* = −0.67; *P* < 0.01), BMI (*r* = −0.66; *P* < 0.01), waist circumference (*r* = −0.40; *P* < 0.01), total calories (*r* = −0.63; *P* < 0.001), fat calories (−0.40; *P* < 0.01), and intakes and changes of dietary consumption of cooking and seasoning oils (−0.59; *P* < 0.001). Moreover, a negative correlation was found with fasting blood glucose (*r* = −0.38; *P* < 0.01), HOMA-IR (*r* = −0.27; *P* < 0.05), total cholesterol (*r* = −0.60; *P* < 0.05), and triglycerides (*r* = −0.31; *P* < 0.05), while a positive correlation was observed with changes of serum adiponectin (*r* = 0.44; *P* < 0.01).

A multiple regression analysis showed that percent changes in body weight (*β* = − 0.37; *t* = − 2.7; *P* = 0.009) and in serum adiponectin (*β* = 0.31; *t* = 2.28; *P* = 0.027) were the only independent predictors of Vitamin E/cholesterol ratio variation after controlling for age, gender, and changes of HOMA-IR, urinary 8-iso-PGF2*α*, and serum sNOX2-dp (adjusted *R* ^2^ = 0.31). Conversely, weight loss (*B* = −0.39; *t* = − 3.0; *P* = 0.004) and changes of urinary 8-iso-PGF2*α* (*B* = − 0.33; *t* = − 2.6; *P* = 0.01) and of serum Vitamin E/cholesterol ratio (*B* = 0.31; *t* = 2.5; *P* = 0.015) were the independent predictors of adiponectin variation (adjusted *R* ^2^ = 0.46).

## 4. Discussion

The study provides evidence that patients with MetS have low serum vitamin E/cholesterol ratio, which is partly related to body weight. Thus, our dietary intervention study demonstrated that weight loss was associated with increased levels of two antioxidant molecules, namely, vitamin E and adiponectin, coincidentally with the downregulation of isoprostane formation and NOX2 activation. These findings suggest that such increase was likely to reflect reduced consumption of molecules with antioxidant property secondary to oxidative stress lowering.

Evidence is mounting that abdominal obesity and MetS are states of chronic oxidative stress [8–10, 12]. Several possible pathogenetic mechanisms have been suggested, including chronic inflammation, metabolic alterations, excessive endothelial ROS production, inadequate antioxidant defences, and lower dietary intake of antioxidants. Indeed, in MetS, increased oxidative stress together with a decreased antioxidative defence seems to contribute to decreased insulin sensitivity [10, 16].

Oxidative stress is brought about by an increase in the production of reactive oxygen species (ROS) and/or a decrease in antioxidant defence. Enhanced production of ROS is likely to occur via upregulation of NOX2, which, in fact, is overactivated in patients with MetS [12]. Adults with MetS have also suboptimal blood and tissue concentration of antioxidants [9]. These include antioxidant enzymes and a broad spectrum of plant-based nutrients (vitamins A, E, C; phytochemicals; and trace elements) that work synergistically to prevent free radical and ROS production [9]. Alpha-tocopherol is the most biologically active form of vitamin E, a fat-soluble antioxidant that stops the production of ROS formed when fat undergoes oxidation. Nuts, seeds, and vegetable oils are among the best sources of alpha-tocopherol [14]. However, in the Italian diet, olive oil and, to a lesser extent, seed oils are the major dietary sources of vitamin E and, recently, a greater adherence to a Mediterranean dietary pattern was found to increase the circulating plasma levels of vitamin E [25].

To the best of our knowledge, there are only two studies reporting vitamin E values in patients with MetS, with controversial results. The first [19] reports that a low vitamin E status characterizes MetS, after adjustment for lipid profile the other one [26] does not show any significant relationship between unadjusted circulating vitamin E and MetS. Furthermore, clinical determinants of vitamin E, as well as its interplay with oxidative stress, were still to be clarified.Our study is in agreement with the presence of low serum vitamin E levels in MetS patients, as compared to controls. This finding is further demonstrated by the close negative association between the number of MetS components and the levels of serum vitamin E/cholesterol ratio. These results are also in agreement with the lineal trend between biomarkers of oxidative stress and increasing number of features of MetS recently reported by Jialal et al. [27].

To further explore the relationship between vitamin E and MetS we investigated the effect of a six-month, low-calorie, dietary intervention on biomarkers of antioxidant status and oxidative stress in patients with the metabolic syndrome. In a previous paper referring to the same patients with MetS [12], we reported that dietary modifications based on moderate calorie restriction are associated with multiple health benefits, including a better control of central obesity, insulin resistance, blood pressure, and atherogenic dyslipidemia, which may be related to a decrease in the production of ROS. In particular, in these patients, moderate weight loss was able to induce a downregulation of NOX2 activation, with a consequent reduction of oxidative stress as assessed by the urinary excretion of isoprostanes, the end products of polyunsaturated fat peroxidation, currently considered the best available biomarkers of lipid peroxidation.

The present study extends these findings demonstrating that moderate weight loss is associated not only to oxidative stress lowering, but also with an increase of antioxidant status. In fact, mean serum Vitamin E/cholesterol ratio raised by 130% in the group of patients achieving at least 5% reduction of body weight. The increase of vitamin E was coincident with the increase of serum adiponectin, another molecule with antioxidant property [28, 29]. Together, these data suggest that weight loss may promote a decrease of oxidative stress with a consequent spared consumption of antioxidants such as vitamin E and adiponectin. 

This finding is of potentially clinical relevance as antioxidants have positive effects on metabolic and glycaemic control. Accordingly, an association was also observed between changes of Vitamin E/cholesterol ratio and changes of indexes of glycemic and lipid metabolism, which is in agreement with the hypothesis that oxidative stress may negatively influence glycaemic control by increasing insulin resistance [30, 31]. Finally, the positive impact of weight loss in enhancing vitamin E was reinforced by the inverse association between the increase of vitamin E/cholesterol ratio and changes of parameters of adiposity–body weight, BMI, and waist circumference.

In our study we may exclude an increased dietary intake of alpha-tocopherol after weight loss. In fact, major dietary changes consisted in the reduction of olive oil and seasoning fat consumption, which lead to an estimated decrease of vitamin E intake from 39.3 to 16.2 mg/day, which is similar to the reported daily intake of healthy, nonobese Italians on a Mediterranean diet [25].

We acknowledge that our study may have some limitations. Lack of randomization to two different diets could have biased the results. We decided, however, that randomizing to two diets, one with and another without calorie restriction, was unethical. An interesting alternative chance was provided by previous studies showing that in case of dietary restriction about 45% of subjects are poor responders to diet [31] and do not achieve a successful weight loss and may therefore be considered a valuable group for comparisons. Based on this assumption, we calculated a sample size that could be adequate for our study hypothesis [12]. Furthermore, we tried to minimize the open design of the intervention study by performing all laboratory analyses in blind.

In conclusion, our findings give further support to the concept that in MetS moderate weight loss is associated with multiple health benefits, some of which are related to an improvement in antioxidant status and a decrease of oxidative stress. Therefore, it is tempting to speculate that in this clinical setting the increase in antioxidant defences may not only reverse ROS generation (and atherosclerosis progression), but also improve insulin sensitivity and lipoprotein profile, thus increasing protection towards diabetes and cardiovascular disease.

Finally, we confirm the importance of calorie restriction as the first-line intervention to reduce the cardiometabolic risk in patients with MetS.

## Figures and Tables

**Table 1:** Clinical characteristics of patients with and without metabolic syndrome.

Variables	Metabolic syndrome	Controls	*P*
	*n* = 92	*n* = 80	
Age (yr)*	55 ± 10.9	54.12 ± 11.75	n.s.
Gender, M/F	57/35	47/33	n.s.
High blood pressure**	90% (*n* = 83)	20% (*n* = 16)	<0.001
Hypertriglyceridemia**	68% (*n* = 63)	12% (*n* = 10)	<0.001
Hyperglycaemia**	43% (*n* = 40)	6% (*n* = 5)	<0.001
Low HDL cholesterol**	58% (*n* = 54)	31% (*n* = 25)	<0.001
Central obesity**	94% (*n* = 87)	16% (*n* = 13)	<0.001
Total cholesterol, mg/dL*	227 ± 53	190 ± 34	<0.001
HDL cholesterol, mg/dL*	45.4 ± 12.4	50.6 ± 14.3	0.01
Triglyceridemia,***	155 (109.7–197.7)	102.0 (84.2–124.5)	<0.001
Vitamin E/cholesterol ratio	4.5 ± 1.2	5.2 ± 0.8	<0.001
Vitamin E *μ*mol/L	26.0 ± 6.7	25.4 ± 5.4	n.s.
Diabetes	22% (*n* = 20)	1% (*n* = 1)	<0.001

*Data are expressed as mean ± SD;

**Defined according to modified ATPIII criteria [4];

***Median and interquartile range.

**Table 2:** Clinical characteristics of patients with serum vitamin E/cholesterol ratio < or ≥5.

Variables	Vitamin E/cholesterol ratio <5 (*n* = 101)	Vitamin E/cholesterol ratio ≥5 (*n* = 71)	*P*
Metabolic syndrome	64% (*n* = 65)	38% (*n* = 27)	0.001
Age (yr)*	54.7 ± 10.6	54.4 ± 12.3	n.s.
Gender, M/F	63/38	41/30	n.s.
High blood pressure**	69% (*n* = 70)	41% (*n* = 29)	<0.001
Hypertriglyceridemia**	49% (*n* = 50)	31% (*n* = 22)	0.019
Hyperglycaemia**	41% (*n* = 41)	14% (*n* = 10)	<0.001
Low HDL cholesterol**	51% (*n* = 52)	35% (*n* = 25)	0.043
Central obesity**	69% (*n* = 70)	41% (*n* = 29)	<0.001
Diabetes	17% (*n* = 17)	6% (*n* = 4)	0.033

*Data are expressed as mean ± S D; **Defined according to modified ATPIII criteria [4].

**Table 3:** Baseline values of some nutritional, anthropometric, and biochemical characteristics of the study participants and changes after 6 months of low-calorie_,_ low-fat diet according to body weight reduction: group A ≥5%; group B <5%.

Variables	Group A			Group B			Between-groups comparison	
	Body weight reduction ≥5%, *N* = 23			Body weight reduction <5%, *N* = 30			of change	
	Baseline	6 months	Δ%	Baseline	6 months	Δ%	Difference (95% CI)	*P* value at 6 months
Total energy (Kcal)	2801 ± 573	2156 ± 364***	−23.0	2952 ± 405	2870 ± 455	−2.7	−562 (−750 to −374)	<.001
Total fat (g)	131.0 ± 28.9	87.4 ± 20.9***	−33.2	133.4 ± 24.5	126.7 ± 25.5	−5.0	−36.8 (−53.1 to −20.5)	<.001
Saturated fat (g)	37.8 ± 10.1	25.2 ± 6.0***	−33.0	41.7 ± 7.2	39.7 ± 8.4	−4.5	−10.6 (−16.0 to −5.1)	<.001
Monounsaturated fat (g)	77.0 ± 18.2	50.0 ± 14.4***	−35.0	74.8 ± 17.1	70.7 ± 16.3	−5.3	−22.9 (−43.1 to −11.8)	<.001
Polyunsaturated fat (g)	11.8 ± 2.3	8.6 ± 2.0***	−26.2	11.4 ± 2.4	11.2 ± 2.3	−1.7	−2.8 (−4.2 to −1.5)	<.001
Oil/seasoning fat (teasp/wk)	147.4 ± 50.2^§^	60.9 ± 11.0***	−58.6	182.0 ± 32.4	177.3 ± 36.1	−2.5	−81.8 (−108.2 to −55.4)	<.001
Body weight, (kg)	96.05 ± 18.3	89.5 ± 17.6***	−6.8	98.7 ± 18.2	98.8 ± 18.2	+0.1	−6.6 (−8.1 to −5.0)	<.001
Urinary 8-isoprostanes (pg/mg creatinine)	144.1 ± 55.3	87.8 ± 53.6**	−39.0	124.5 ± 63.3	119.6 ± 58.9	−3.9	−51.3 (−99.0 to −3.6)	.035
sNOX2-dp (pg/mL)	42.2 ± 22.7	32.8 ± 24.6*	−22.2	45.0 ± 17.0	41.0 ± 17.5	−8.8	−20.2 (−34.5 to 5.9)	<.01
Adiponectin (ng/mg)	4.3 ± 1.0	9.7 ± 1.9***	+125.5	4.5 ± 1.0	5.1 ± 1.3	13.3	+4.8 (3.6 to 6.0)	<.01
Vitamin E (*μ*mol)	21.4 ± 7.3	35.2 ± 8.4***	+100.2	17.3 ± 5.6	17.6 ± 5.4	+4.3	−95.4 (−150.8 to 41.0)	<.001
Cholesterol (*μ*mol)	5.6 ± 1.0	4.8 ± 0.9**	−13.0	5.4 ± 0.8	5.4 ± 0.6	−0.1	13.1 (6.7 to 19.6)	<.001
Vitamin E/cholesterol ratio	4.8 ± 1.7	9.4 ± 2.9***	+129.8	4.3 ± 1.0	4.1 ± 1.2	+5.4	+4.8 (3.5 to 6.1)	<.001

*P < .05; **P < .01; ***P < .001, 6 months versus baseline.

^§^ *P* < .05 versus group B at baseline.

Statistical analysis was performed by *t*-test (for between-groups analysis) and paired *t*-test (for within-group analysis).

**Table 4:** Correlations between changes in serum vitamin/cholesterol ratio and changes of some nutritional, antropometric, metabolic and oxidative stress parameters.

	Vitamin E/chol. ratio	Weight	BMI	Waist circ.	Total calories	Fat calories	Oil intake	Blood glucose	HOMA-IR	Tot. Choi	Trigl.	Adipo.	NOX2	Urinary 8-iso-PGF2*α*
Vitamin E/chol. ratio	1.00	—	—	—	—	—	—	—	—	—	—	—	—	—
Weight (kg)	−.67**	1.00	—	—	—	—	—	—	—	—	—	—	—	—
BMI	−.66**	.98***	1.00	—	—	—	—	—	—	—	—	—	—	—
Waist circle (cm)	−.40**	.73***	.77***	1.00	—	—	—	—	—	—	—	—	—	—
Total calories	−.63***	.87***	.86***	.60***	1.00	—	—	—	—	—	—	—	—	—
Fat calories	−.40**	.58***	.57***	.39**	.67***	1.00	—	—	—	—	—	—	—	—
Dietary oil intake (tablesp/day)	−.59***	.75***	.76***	.53***	.74***	.60***	1.00	—	—	—	—	—	—	—
Blood glucose (mg/dL)	−.38**	.45***	.47***	.47***	.37**	−.03	.49***	1.00	—	—	—	—	—	—
HOMA-IR	−.27*	.34*	.37**	.25	.20	−.02	.24	.41**	1.00	—	—	—	—	—
Total cholesterol (mg/dL)	−.60*	.57***	.58***	.46***	.60***	.37**	.46**	.23	.31*	1.00	—	—	—	—
Triglycerides (mg/dL)	−.31*	.36**	.34*	.26	.31*	.10	.24	.14	.30*	.56***	1.00	—	—	—
Adiponectin (ng/mg)	.44**	−.52***	−.49***	−.40***	−.39**	−.29*	−.45***	−.31*	−.06	−.363**	−.07	1.00	—	—
sNOX2-dp (pg/mL)	−.13	.30*	.27*	.21	.25	.07	.17	.26	.11	-.01	−.07	.38***	1.00	—
Urinary 8-iso-PGF2*α* (pg/mg creatinine)	−.14	.31*	.29*	.31*	.26	.09	.15	.17	.22	.07	−.07	−.43***	.63***	1.00

*P < .05; **P < .01; ***P < .001.
